# Secukinumab in non-radiographic axial spondyloarthritis: subgroup analysis based on key baseline characteristics from a randomized phase III study, PREVENT

**DOI:** 10.1186/s13075-021-02613-9

**Published:** 2021-09-04

**Authors:** Jürgen Braun, Ricardo Blanco, Helena Marzo-Ortega, Lianne S. Gensler, Filip van den Bosch, Stephen Hall, Hideto Kameda, Denis Poddubnyy, Marleen van de Sande, Anna S. Wiksten, Brian O. Porter, Abhijit Shete, Hanno B. Richards, Sibylle Haemmerle, Atul Deodhar

**Affiliations:** 1 Rheumazentrum Ruhrgebiet Herne, Ruhr-University Bochum, Bochum, Germany; 2 IDIVAL, Hospital University Marqués de Valdecilla, Santander, Spain; 3 NIHR Leeds Biomedical Research Centre, Leeds Teaching Hospitals NHS Trust and LIRMM, University of Leeds, Leeds, UK; 4 Department of Medicine/Rheumatology, University of California, San Francisco, San Francisco, CA USA; 5 Department of Internal Medicine and Pediatrics, VIB Center for Inflammation Research, Ghent University, Ghent, Belgium; 6 Monash University, Melbourne, Australia; 7 Toho University, Tokyo, Japan; 8 Charité Universitätsmedizin, Berlin, Germany; 9 Department of Rheumatology and Clinical Immunology, Amsterdam Infection & Immunity Institute, Amsterdam UMC, AMC/University of Amsterdam, Amsterdam, The Netherlands; 10 Amsterdam Rheumatology and Immunology Centre (ARC), Amsterdam, The Netherlands; 11 Novartis Pharma AG, Basel, Switzerland; 12 Novartis Pharmaceuticals Corporation, East Hanover, USA; 13 Oregon Health & Science University, Portland, USA

**Keywords:** Non-radiographic axial spondyloarthritis, C-reactive protein, Magnetic resonance imaging, Interleukins, Biologicals, Human leukocyte antigen B27, Gender

## Abstract

**Background:**

To investigate the efficacy of secukinumab in patients with active non-radiographic axial spondyloarthritis (nr-axSpA) grouped by disease activity as assessed by C-reactive protein (CRP) levels and/or magnetic resonance imaging (MRI) scores, human leukocyte antigen (HLA)-B27 status, and sex.

**Methods:**

The phase III PREVENT study randomized (1:1:1) 555 patients to receive subcutaneous secukinumab 150 mg with (LD) or without (NL) loading dose or placebo weekly, followed by every 4 weeks starting at week 4. Here, we report the results of a post hoc analysis reporting the efficacy outcomes (pooled secukinumab) to 16 weeks by CRP, MRI, HLA-B27, and sex.

**Results:**

Efficacy differences between the secukinumab and the placebo groups were highest in the CRP+, MRI+, HLA-B27+, and male subgroups, particularly for Ankylosing Spondylitis Disease Activity Score-CRP inactive disease and Assessment of SpondyloArthritis international Society (ASAS) partial remission outcomes. ASAS40 response rates in the CRP+/MRI+ subgroup was 52.3% (secukinumab) versus 21.8% (placebo; *P* < 0.0001) at week 16. ASAS40 response rates (secukinumab versus placebo) were 43.9% versus 32.6% in HLA-B27+, 32.7% versus 16.4% in HLA-B27− subgroups, 51.2% versus 30.8% in male, and 31.7% versus 25.3% in female patients, respectively.

**Conclusions:**

Secukinumab improved the signs and symptoms of nr-axSpA across patients grouped by CRP (+/−) and/or MRI (+/−) status, HLA-B27 (+/−) status, and sex. The highest treatment differences between secukinumab and placebo were observed in patients with both elevated CRP and evidence of sacroiliitis on MRI. Treatment difference was minimal between HLA-B27 (+) and (−) subgroups. Male patients had higher relative responses than female patients.

**Trial registration:**

ClinicalTrials.gov, NCT02696031. Registered on 02 March 2016

**Supplementary Information:**

The online version contains supplementary material available at 10.1186/s13075-021-02613-9.

## Background

Axial spondyloarthritis (axSpA) is a chronic inflammatory disease, with an estimated prevalence of no less than 0.5% in the global population [1]. AxSpA is categorized into radiographic axSpA (r-axSpA) or ankylosing spondylitis (AS) and non-radiographic axSpA (nr-axSpA) based on the presence of definite structural changes on radiographs of the sacroiliac joints (SIJs). Nr-axSpA may progress to r-axSpA or AS over the course of the disease. Spinal inflammation can be visualized using magnetic resonance imaging (MRI) and new bone formation using conventional radiography [2–8]. Patients with nr-axSpA can show a comparable disease activity and burden as patients with r-axSpA or AS. The rate of progression from nr-axSpA to AS varies over the years, with a lifetime risk of progression of approximately 50% [8–13].

The presence of objective signs of inflammation (elevated C-reactive protein [CRP] levels and/or evidence of sacroiliitis on MRI) is important prognostic indicators for nr-axSpA, as patients with elevated CRP levels and/or positive MRI findings are more likely to develop definite radiographic changes in a later stage of the disease [8, 9, 13–15]. Clinical assessments of disease activity and predictors of good response to treatment are essential for the management of nr-axSpA, irrespective of the presence or absence of radiographic changes [5, 13, 15]. Other predisposing factors include human leukocyte antigen (HLA)-B27 and sex. HLA-B27 positivity is a predictor of radiographic progression in nr-axSpA, with male patients more prone to disease progression [3, 7–10].

Non-steroidal anti-inflammatory drugs (NSAIDs) are used as first-line therapy in patients with axSpA. Tumor necrosis factor inhibitors (TNFi) are recommended in patients with active axSpA and objective signs of inflammation despite treatment with NSAIDs [15, 16]. In the updated Assessment of SpondyloArthritis international Society and European League Against Rheumatism (ASAS-EULAR) and the American College of Rheumatology, Spondyloarthritis Research and Treatment Network and Spondylitis Association of America (ACR-SPARTAN-SAA) treatment recommendations, interleukin (IL)-17 inhibitors are recommended in patients with axSpA in case of primary non-response to TNFi [15, 16]. Secukinumab, a human monoclonal antibody that directly inhibits IL-17A, has demonstrated sustained improvement in the signs and symptoms of AS over 5 years [17–19]. PREVENT is a phase III study of secukinumab in patients with active nr-axSpA [20]. In this study, secukinumab 150 mg significantly improved the signs and symptoms of nr-axSpA through week 52. Abnormal CRP levels and MRI evidence of inflammation in the SIJs at baseline may influence the efficacy of biologics in patients with axSpA, along with HLA-B27 status and sex [15, 21–31]. Elevated CRP, MRI evidence of SIJ inflammation, HLA-B27 positivity, and male sex are known predictors of better treatment response to biologic disease-modifying anti-rheumatic drugs (bDMARDs) [21–31]. It is therefore of particular interest to further elucidate the influence of these factors on the efficacy of secukinumab. Here, we report the results of an exploratory efficacy analysis through week 16 in patients grouped by CRP and/or MRI status (+/−), HLA-B27 status (+/−), and sex (male or female) at screening from the PREVENT study.

## Methods

### Study design

PREVENT (NCT02696031) was a 2-year randomized, double-blind, placebo-controlled phase III study with an extension phase of up to 2 years in patients with nr-axSpA. The study was conducted across 130 sites in 24 countries. Detailed inclusion and exclusion criteria and study design have been reported previously [20].

The study protocol was reviewed and approved by the independent ethics committee or institutional review board for each participating center. The study was conducted according to the International Council for Harmonization E6 Guideline for Good Clinical Practice that has its origin in the Declaration of Helsinki [32]. Written informed consent was obtained from all participants. Data were collected in accordance with the Good Clinical Practice guidelines by the study investigators and analyzed by the sponsor.

### Participants

Patients (aged ≥ 18 years) with active axSpA fulfilling the ASAS classification criteria for nr-axSpA (inflammatory back pain for ≥ 6 months, disease onset at < 45 years of age and sacroiliitis on MRI with ≥ 1 SpA feature or HLA-B27+ status with ≥ 2 SpA features) plus objective signs of inflammation (MRI with SIJ inflammation [by central reading] and/or high-sensitivity CRP [hsCRP] levels of > upper limit of normal [ULN; hsCRP of > 5 mg/L as defined by the central laboratory]) were included. This eligibility criterion for MRI was based on a qualitative assessment (yes or no) from a single centralized read at baseline. At randomization, patients were stratified according to the objective signs of inflammation based on their CRP and MRI status (CRP+/MRI+, CRP+/MRI−, and CRP−/MRI+) at screening. CRP+ was defined as a value above ULN (hsCRP of > 5 mg/L) as determined by the central laboratory. MRI+ was defined by the presence of inflammatory lesions on the SIJs according to the ASAS-Outcome Measures in Rheumatology Clinical Trials (OMERACT) definition as assessed by a central reader [33].

The MRI scoring for the SIJ images was performed as defined in the imaging charter for the PREVENT study. As part of the screening process, the SIJ images were assessed by one of three reading experts assigned to the study for signs of inflammation. For the efficacy assessments, the SIJs were assessed according to the validated Berlin Active Inflammatory Lesions Scoring by two fully blinded independent central readers; the most discrepant cases (10%) were adjudicated by a third reader [34]. The mean baseline SIJ edema score using observed data for all patients was 2.5 for the secukinumab 150 mg with loading (150 mg LD) group, 2.1 for the secukinumab 150 mg without loading (150 mg NL) group, and 2.5 for the placebo group, thus ~ 10% of the total score (Berlin Active Inflammatory Lesions Scoring of 0–24). The median of the scores was ~ 2, and a cutoff of ≥ 2 was therefore used for the further subgroup analysis of patients with positive MRI findings at baseline.

Patients previously treated with a TNFi (no more than one) could participate if they had an inadequate response or were intolerant. Patients could continue to receive the following medications at a stable dose: sulfasalazine (≤ 3 g/day), methotrexate (≤ 25 mg/week), corticosteroids (≤ 10 mg/day prednisone or equivalent), and NSAIDs.

The key exclusion criteria included evidence of radiographic sacroiliitis per the modified New York criteria for AS (assessed centrally) and active ongoing inflammatory conditions other than axSpA. Details of the eligibility criteria have been published previously [20].

### Interventions

Eligible patients were randomly (1:1:1) allocated to receive subcutaneous (s.c.) secukinumab 150 mg LD, s.c. secukinumab 150 mg NL, or placebo at baseline and weeks 1, 2, and 3, followed by every 4 weeks starting at week 4. The 150 mg NL group received placebo at weeks 1, 2, and 3 to maintain blinding. The treatment blinding for all investigators, site personnel, and patients was maintained until week 52.

### Efficacy assessments

Exploratory efficacy assessments at week 16 included ASAS40, Bath Ankylosing Spondylitis Disease Activity Index (BASDAI) 50, ASAS partial remission (PR), and Ankylosing Spondylitis Disease Activity Score-CRP (ASDAS-CRP) inactive disease (ID) response rates in the overall population independently grouped by MRI (+/−) status, CRP (+/−) status, HLA-B27 (+/−) status, and sex (male or female) at screening. Treatment difference in ASAS40 responses between secukinumab and placebo was assessed in patients grouped by MRI status (+/−), CRP status (+/−), HLA-B27 status (+/−), and sex (male or female) at screening. Efficacy assessments according to the randomization stratification (CRP+/MRI+, CRP+/MRI−, and CRP−/MRI+ at screening) included ASAS40, BASDAI50, ASAS PR, and ASDAS-CRP ID response rates and the mean change from baseline in total BASDAI and Bath Ankylosing Spondylitis Functional Index (BASFI) scores through week 16. The proportion of patients (male versus female) achieving ASAS40, BASDAI50, ASAS PR, and ASDAS-CRP ID responses was assessed for the subgroups through week 16. In addition, ASAS40, ASAS PR, and ASDAS-CRP ID response rates at week 16 were also assessed in the CRP−/MRI+ subgroup by screening the SIJ MRI scores (< 2 or ≥ 2).

### Statistical analysis

Data for secukinumab are presented pooled (150 mg LD plus NL) versus placebo up to week 16. Subgroup analyses based on CRP, MRI, HLA-B27 status, and sex at screening are exploratory, and only the percentage response or the mean change from baseline data are presented. While the analyses were not powered for formal statistical testing, inferential statistics and estimated means and proportions with 95% confidence intervals (CIs) are presented for the interest of the reader. Notably, all *P* values presented are unadjusted for multiplicity. Analyses were performed on the full analysis set, which comprised all patients who were randomized and had study treatment assigned. The details of the sample size calculation and statistical analysis have been reported previously [20]. Missing values were imputed as non-responders for binary variables and via mixed-effects model repeated measures (valid under the missing at random assumption) for continuous variables up to week 16.

## Results

Demographic and baseline disease characteristics were comparable across the treatment groups and reported previously [20]. At baseline, 54.1% (300/555) of the patients were female, 68.8% (382/555) were HLA-B27+, 56.9% (316/555) had elevated hsCRP levels (≥ 5 mg/L), and 73% (405/555) had SIJ inflammation on MRI. According to the stratification, 29.9% (166/555) of the overall population was CRP+/MRI+, 27.7% (154/555) was CRP+/MRI−, and 42.3% (235/555) was CRP−/MRI+. The median (minimum–maximum) hsCRP level (mg/L) was 12.2 (7.5–31.9) in the CRP+/MRI+ subgroup, 11.4 (7.7–28.4) in the CRP+/MRI− subgroup, and 2.2 (1.2–3.7) in the CRP−/MRI+ subgroup. The proportion of male and female patients by screening MRI scores (< 2 or ≥ 2) were 46.7% (< 2; 49/105) and 56.2% (< 2; 73/130), and 53.3% (≥ 2; 56/105) and 43.9% (≥ 2; 57/130), respectively, in the CRP−/MRI+ subgroup. The number of patients in the pooled secukinumab versus placebo group by sex for all subgroups is presented in Supplement Table S1. By week 24, 5% (28/555) of the patients had missing data.

The primary and all key secondary endpoints at week 16 were met and have been reported previously [20]. The proportion of patients achieving ASAS40, BASDAI50, ASAS PR, and ASDAS-CRP ID responses in the pooled secukinumab group versus the placebo group across the individual MRI, CRP, HLA-B27, and sex subgroups are shown in Table 1, with the highest responses observed in the CRP+, MRI+, HLA-B27+, and male subgroup at week 16.

**Table 1:** Key efficacy outcomes by independent subgroups at screening analyzed at week 16

Endpoints, % responders (*n*/*M*)	Subgroups	Pooled secukinumab 150 mg	Placebo
**ASAS40**	CRP+	44.5^§^ (94/211)	26.7 (28/105)
	CRP−	34.8 (55/158)	29.6 (24/81)
	MRI+	42.9^§^ (114/266)	27.3 (38/139)
	MRI−	34.0 (35/103)	29.8 (14/47)
	HLA-B27+	43.9^‡^ (111/253)	32.6 (42/129)
	HLA-B27−	32.7^‡^ (37/113)	16.4 (9/55)
	Male	51.2^§^ (84/164)	30.8 (28/91)
	Female	31.7 (65/205)	25.3 (24/95)
**BASDAI50**	CRP+	40.3^*^ (85/211)	19.0 (20/105)
	CRP−	33.5 (53/158)	23.5 (19/81)
	MRI+	38.7^*^ (103/266)	19.4 (27/139)
	MRI−	34.0 (35/103)	25.5 (12/47)
	HLA-B27+	41.5^†^ (105/253)	24.8 (32/129)
	HLA-B27−	29.2^§^ (33/113)	10.9 (6/55)
	Male	45.1^§^ (74/164)	26.4 (24/91)
	Female	31.2^§^ (64/205)	15.8 (15/95)
**ASAS PR**	CRP+	23.7^*^ (50/211)	6.7 (7/105)
	CRP−	18.4^§^ (29/158)	7.4 (6/81)
	MRI+	21.4^*^ (57/266)	6.5 (9/139)
	MRI−	21.4^‡^ (22/103)	8.5 (4/47)
	HLA-B27+	25.3^*^ (64/253)	8.5 (11/129)
	HLA-B27−	13.3^‡^ (15/113)	3.6 (2/55)
	Male	28.7^*^ (47/164)	9.9 (9/91)
	Female	15.6^†^ (32/205)	4.2 (4/95)
**ASDAS-CRP ID**	CRP+	20.9^*^ (44/211)	3.8 (4/105)
	CRP−	21.5 (34/158)	13.6 (11/81)
	MRI+	23.7^*^ (63/266)	8.6 (12/139)
	MRI−	14.6 (15/103)	6.4 (3/47)
	HLA-B27+	24.5^*^ (62/253)	9.3 (12/129)
	HLA-B27−	14.2 (16/113)	5.5 (3/55)
	Male	29.9^*^ (49/164)	9.9 (9/91)
	Female	14.1^‡^ (29/205)	6.3 (6/95)

NRI data presented for all variables

*ASAS* Assessment of SpondyloArthritis international Society, *ASDAS* Ankylosing Spondylitis Disease Activity Score, *BASDAI* Bath Ankylosing Spondylitis Disease Activity Index, *CRP* C-reactive protein, *HLA* human leukocyte antigen, *ID* inactive disease, *M* number of evaluable patients, *MRI* magnetic resonance imaging, *NRI* non-responder imputation, *PR* partial remission

^*^P < 0.0001, ^†^*P* < 0.001, ^§^*P* < 0.01, ^‡^*P* < 0.05 versus placebo

**Fig. 1:**
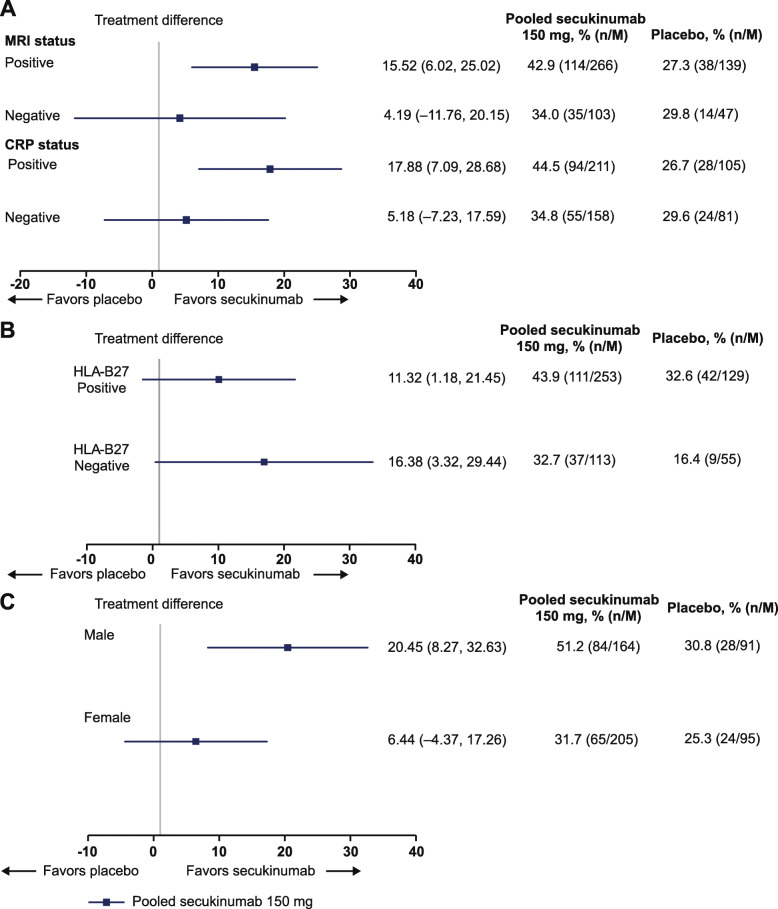
Differences in the ASAS40 response between secukinumab and placebo at week 16. **A** MRI and CRP status. **B** HLA-B27 status. **C** sex at screening. ASAS, Assessment of SpondyloArthritis international Society; CRP, C-reactive protein; HLA, human leukocyte antigen; *M*, number of evaluable patients; MRI, magnetic resonance imaging

Absolute treatment differences in ASAS40 responses at week 16 between pooled secukinumab and placebo by MRI, CRP, HLA-B27 status, and by sex are shown in Fig. 1. Treatment differences between secukinumab and placebo were highest in patients with MRI+ and CRP+ at screening (Fig. 1A). Treatment responses between secukinumab and placebo were comparable in patients who were HLA-B27− compared with those who were HLA-B27+ at screening with overlapping CIs (Fig. 1B). Treatment differences between secukinumab and placebo were higher in male patients compared with female patients (Fig. 1C).

**Fig. 2:**
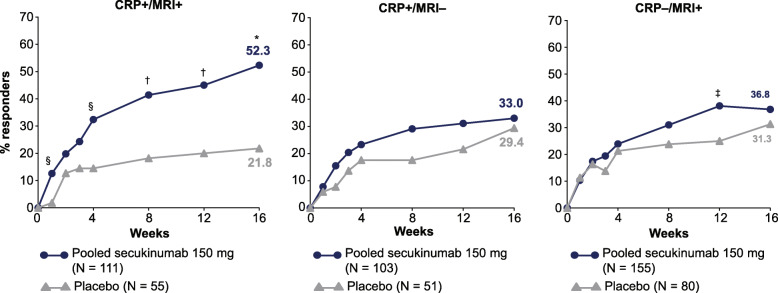
Proportion of patients achieving ASAS40 response in the overall population through week 16. *P < 0.0001, ^†^*P* < 0.001, ^§^*P* < 0.01, ^‡^*P* < 0.05 versus placebo. Data presented as NRI through week 16. ASAS, Assessment of SpondyloArthritis international Society; CRP, C-reactive protein; MRI, magnetic resonance imaging; *N*, total number of patients; NRI, non-responder imputation

ASAS40 response at week 16 in the overall population who were CRP+/MRI+ at screening in the pooled secukinumab group was 52.3% compared with 21.8% (*P* < 0.0001) in the placebo group (Fig. 2). Corresponding response rates in the other two subgroups were 33.0% versus 29.4% (CRP+/MRI−) and 36.8% versus 31.3% (CRP−/MRI+) in the pooled secukinumab group versus the placebo group, respectively. ASAS40 response rates (pooled secukinumab versus placebo) by sex were broadly similar to the response rates observed in the overall population, with slightly lower responses in female patients (Supplement Table S2).

**Fig. 3:**
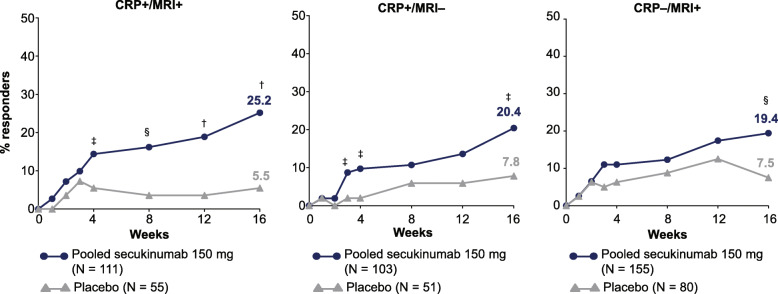
Proportion of patients achieving ASAS PR response through week 16. ^†^*P* < 0.001, ^§^*P* < 0.01, ^‡^*P* < 0.05 versus placebo. Data presented as NRI through week 16. ASAS, Assessment of SpondyloArthritis international Society; CRP, C-reactive protein; MRI, magnetic resonance imaging; *N*, total number of patients; NRI, non-responder imputation; PR, partial remission

ASAS PR response rates and additional outcome measures (BASDAI50 and ASDAS-CRP ID) across the three subgroups (CRP+/MRI+, CRP+/MRI−, and CRP−/MRI+) are shown in Fig. 3 and Supplement Table S3, respectively, with the greatest differences between the pooled secukinumab and placebo observed in the CRP+/MRI+ subgroup across the outcome variables. The most notable treatment differences between secukinumab and placebo were observed for ASAS PR and ASDAS-CRP ID.

**Fig. 4:**
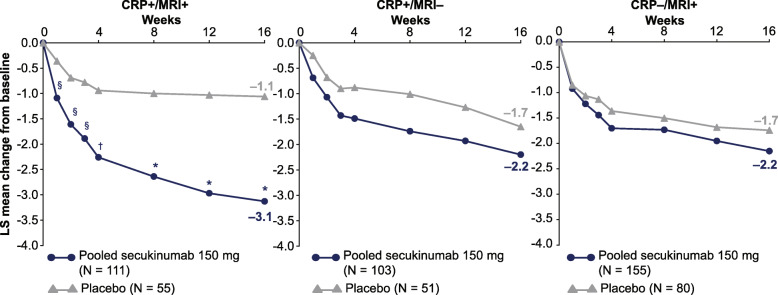
Improvement in BASDAI score through week 16. *P < 0.0001, ^†^*P* < 0.001, ^§^*P* < 0.01 versus placebo. Data presented as MMRM through week 16. BASDAI, Bath Ankylosing Spondylitis Disease Activity Index; CRP, C-reactive protein; LS, least squares; MMRM, mixed-effects model repeated measures; MRI, magnetic resonance imaging; *N*, total number of patients

The mean change from baseline in BASDAI and BASFI scores at week 16 are shown in Fig. 4 and Supplement Table S3, respectively, with the greatest treatment differences between the pooled secukinumab and placebo observed in the CRP+/MRI+ subgroup. The proportion of patients (male versus female) achieving ASAS40, BASDAI50, ASAS PR, and ASDAS-CRP ID responses at week 16 across all subgroups are presented in Supplement Table S2. The proportion of patients achieving ASAS40, ASAS PR, and ASDAS-CRP ID responses at week 16 in the CRP−/MRI+ subgroup by screening SIJ MRI scores (< 2 or ≥ 2) are presented in Table 2. Those patients with a score of ≥ 2 consistently had higher efficacy responses to secukinumab, while responses to placebo were not affected by the baseline SIJ MRI score.

**Table 2:** Key efficacy outcomes at week 16 in the CRP−/MRI+ subgroup by screening SIJ MRI score

Endpoints, % responders (*n*/*M*)	Screening SIJ MRI score	Pooled secukinumab 150 mg	Placebo
**ASAS40**	< 2	31.7 (26/82)	30.0 (12/40)
	≥ 2	42.5 (31/73)	32.5 (13/40)
**ASAS PR**	< 2	14.6 (12/82)	7.5 (3/40)
	≥ 2	24.7^§^ (18/73)	7.5 (3/40)
**ASDAS-CRP ID**	< 2	17.1 (14/82)	15.0 (6/40)
	≥ 2	30.1^‡^ (22/73)	12.5 (5/40)

NRI data presented for all variables

The total number of patients in the screening MRI score of < 2 subgroup was 82 (pooled secukinumab 150 mg) and 40 (placebo) and in the screening MRI score ≥ 2 subgroup was 73 (pooled secukinumab 150 mg) and 40 (placebo)

*ASAS* Assessment of SpondyloArthritis international Society, *ASDAS* Ankylosing Spondylitis Disease Activity Score, *CRP* C-reactive protein, *ID* inactive disease, *M* number of evaluable patients, *MRI* magnetic resonance imaging, *NRI* non-responder imputation, *PR* partial remission, *SIJ* sacroiliac joint

^§^*P* < 0.01, ^‡^*P* < 0.05 versus placebo

## Discussion

This report of efficacy outcomes from a subgroup analysis of the PREVENT study assessed the efficacy of secukinumab in patients with nr-axSpA across important subgroups of CRP, MRI, and HLA-B27 status and in male and female patients. For the subgroup of patients with MRI+ at screening, the absolute treatment differences in the ASAS40 response between secukinumab and placebo were higher than those in the MRI− subgroup. A similar trend was observed in the CRP subgroups for the treatment differences between secukinumab and placebo in ASAS40 responses, with higher response rates noted for the CRP+ (> 5 mg/L) subgroup compared with those for the CRP− (≤5 mg/L) subgroup.

To further explore the combined influence of MRI+ and CRP+ status, additional efficacy analyses were conducted in patients stratified at randomization based on their CRP and MRI status (CRP+/MRI+, CRP+/MRI−, and CRP−/MRI+). Secukinumab provided numerically higher response rates versus placebo across subgroups, with the greatest treatment differences between secukinumab and placebo observed for ASAS PR and ASDAS-CRP ID. Placebo responses were higher in the single-positive subgroups (CRP+/MRI− or CRP−/MRI+) than in the double-positive subgroup (CRP+/MRI+) for ASAS40 and BASDAI50 up to week 16. Placebo responses were lower when looking at higher-hurdle endpoints such as ASAS PR and ASDAS-CRP ID in the single-positive subgroups (CRP+/MRI− or CRP−/MRI+). The definitive reasons for the high variability in placebo response cannot be speculated from the outcome of this post hoc analysis; however, this may be due to an expectation for the efficacy of biologics, particularly in biologic-naïve patients and owing to the subjective nature of the majority of the outcome measures used in axSpA studies [20]. As the cohort size of each treatment group was limited, data obtained from subgroup analyses for continuous outcomes (BASDAI or BASFI) may offer better interpretability compared with those for binary outcomes. Secukinumab-treated patients showed a consistent trend in terms of mean reduction in BASDAI or BASFI score across CRP/MRI subgroups over time through week 16 versus placebo.

To further validate the outcomes of these exploratory analyses in the single-positive subgroups and to better understand the data, we considered a threshold of MRI positivity. The data analysis based on screening SIJ MRI scores (< 2 or ≥ 2) in patients with a CRP−/MRI+ status suggests that patients with a higher level of inflammation of the SIJs at baseline experience higher relative efficacy compared to those with lesser SIJ inflammation, while placebo responses appear unaffected. This highlights the potential need for a review of the criteria used to evaluate and score SIJ edema by MRI.

According to the ASAS-EULAR treatment guideline, either an elevated CRP or positive MRI may be taken into consideration for bDMARD therapy in patients with axSpA, irrespective of the presence or absence of radiographic sacroiliitis [15]. Earlier studies with TNFi in patients with nr-axSpA have shown that elevated CRP and/or a positive MRI status at baseline had a significant impact on treatment response rates [15, 22, 27–29, 31]. Indeed, an elevated CRP level has been reported to be the strongest predictor and a positive MRI status, the second best predictor of treatment response to TNFi in both patients with r-axSpA and nr-axSpA [15, 22, 27, 28, 31]. In a pooled post hoc analysis from the pivotal MEASURE 1 and 2 studies, secukinumab provided a rapid and sustained improvement over 3 years across multiple clinical domains in patients with r-axSpA or AS irrespective of baseline CRP, with a greater response to treatment reported in patients with elevated CRP [35]. The results from the current post hoc analysis further confirm the importance of these parameters.

The HLA-B27 status has been reported to be a predictor of response to biologic therapy in patients with axSpA. The proportion of HLA-B27 positivity in this nr-axSpA population is 69%, which is slightly lower than previous studies [21, 22, 26–28, 31] and interestingly, ASAS40 responses appeared independent of the HLA-B27 status in the current analysis.

Evidence from other trials suggests that female patients with axSpA derive less efficacy from biologics compared with male patients, even though the disease burden at baseline is comparable or worse in female patients compared with that in male patients [22–25, 31]. In the current analysis, the disease burden was similar (mean baseline BASDAI score was 6.89 in males and 6.94 in females), and HLA-B27 positivity was 73% (185/255) in males and 66% (197/300) in females. The severity of SIJ edema by MRI was greater in males (mean baseline SIJ MRI score ≥ 2 was 53%) than in females (mean baseline SIJ MRI score ≥ 2 was 44%), indicating that males may be more prone to develop structural abnormalities in the course of the disease. While clinically meaningful efficacy was seen in both male and female patients, higher responses were consistently observed in males across all outcome measures. These results are consistent with the observations from other studies and support the recommendation for an individual assessment of sex-independent risk factors in patients with axSpA [22–25, 28, 36–38].

Limitations of these exploratory analyses included a relatively small number of patients in some of the individual subgroups. Thus, the analyses were not powered to derive definitive conclusions, but rather to explore trends as opposed to statistical analyses of significance. Therefore, the results need to be interpreted with caution. It should also be noted that > 90% of the population was TNFi-naïve and that the trial did not enroll patients with a CRP−/MRI− status. Further, the complex interaction between the factors contributing to treatment response is yet to be explored.

## Conclusions

In summary, secukinumab improved the signs and symptoms of nr-axSpA across patient subgroups based on CRP (+/−) and/or MRI (+/−) status, HLA-B27 (+/−) status, and sex. The highest treatment differences between secukinumab and placebo were observed in patients with both elevated CRP levels and evidence of sacroiliitis on MRI, and in male patients, whereas the difference was minimal between HLA-B27 positive and negative subgroups. The results from these exploratory analyses further substantiate the primary and key secondary efficacy outcomes previously reported from the PREVENT study and provide additional evidence supporting the efficacy of secukinumab in patients with axSpA.

## Supplementary Information

**Additional file 1: Table S1.** Number of patients in each subgroup. **Table S2.** Key efficacy endpoints by subgroups and by gender at screening analysed at Week 16. **Table S3.** Additional efficacy endpoints in the subgroups analysed at Week 16.

## Data Availability

The data sets generated during and/or analyzed at the end of the current study are not publicly available. Novartis is committed to sharing with qualified external researchers’ access to patient-level data and supporting clinical documents from eligible studies. These requests are reviewed and approved based on scientific merit. All data provided are anonymized to respect the privacy of patients who have participated in the trial in line with applicable laws and regulations. The data may be requested from the corresponding author of the manuscript.

## Abbreviations

ACR: American College of Rheumatology
AS: Ankylosing spondylitis
ASAS: Assessment of SpondyloArthritis international Society
ASDAS: Ankylosing Spondylitis Disease Activity Score
axSpA: Axial spondyloarthritis
BASDAI: Bath Ankylosing Spondylitis Disease Activity Index
BASFI: Bath Ankylosing Spondylitis Functional Index
bDMARDs: Biologic disease-modifying anti-rheumatic drugs
CIs: Confidence intervals
CRP: C-reactive protein
EULAR: European League Against Rheumatism
HLA: Human leukocyte antigen
hsCRP: High-sensitivity CRP
ID: Inactive disease
IL: Interleukin
LD: Loading
MRI: Magnetic resonance imaging
NL: Without loading
nr-axSpA: Non-radiographic axSpA
NSAIDs: Non-steroidal anti-inflammatory drugs
OMERACT: Outcome Measures in Rheumatology Clinical Trials
PR: Partial remission
r-axSpA: Radiographic axSpA
SAA: Spondylitis Association of America
SIJs: Sacroiliac joints
SPARTAN: Spondyloarthritis Research and Treatment Network
TNFi: Tumor necrosis factor inhibitors
ULN: Upper limit of normal
