# Supplementary material for: Secukinumab in non-radiographic axial spondyloarthritis: subgroup analysis based on key baseline characteristics from a randomized phase III study, PREVENT
Source: Arthritis Res Ther. 2021 Sep 4;23:231. doi: 10.1186/s13075-021-02613-9 (PMC8418044; doi:10.1186/s13075-021-02613-9)
Supplement: Supplementary file 1 — Additional file 1: Table S1. Number of patients in each subgroup. Table S2. Key efficacy endpoints by subgroups and by gender at screening analysed at Week 16. Table S3. Additional efficacy endpoints in the subgroups analysed at Week 16. [file 13075_2021_2613_MOESM1_ESM.docx]

**SUPPLEMENT**

| **Table S1. Number of patients in each subgroup** | | | | |
| --- | --- | --- | --- | --- |
| **Subgroups, n** | **Male** | | **Female** | |
|  | **Pooled secukinumab 150 mg** | **Placebo** | **Pooled secukinumab 150 mg** | **Placebo** |
| **CRP+/MRI+** | 62 | 36 | 49 | 19 |
| **CRP+/MRI−** | 32 | 20 | 71 | 31 |
| **CRP−/MRI+** | 70 | 35 | 85 | 45 |
| **CRP+** | 90 | 54 | 121 | 51 |
| **CRP−** | 74 | 37 | 84 | 44 |
| **MRI+** | 132 | 70 | 134 | 69 |
| **MRI−** | 32 | 21 | 71 | 26 |
| **HLA-B27+** | 122 | 63 | 131 | 66 |
| **HLA-B27–** | 40 | 26 | 73 | 29 |
| CRP, C-reactive protein; HLA, human leukocyte antigen; MRI, magnetic resonance imaging. | | | | |

| **Table S2**. **Key efficacy endpoints by subgroups and by gender at screening analysed at Week 16** | | | | | |
| --- | --- | --- | --- | --- | --- |
| **Endpoints, % responders (n/M)** | **Subgroups** | **Male** | | **Female** | |
|  |  | **Pooled secukinumab 150 mg** | **Placebo** | **Pooled secukinumab 150 mg** | **Placebo** |
| **ASAS40** | CRP+/MRI+ | 54.8^†^ (34/62) | 22.2 (8/36) | 49.0^‡^ (24/49) | 21.1 (4/19) |
|  | CRP+/MRI− | 43.8 (14/32) | 35.0 (7/20) | 28.2 (20/71) | 25.8 (8/31) |
|  | CRP−/MRI+ | 51.4 (36/70) | 37.1 (13/35) | 24.7 (21/85) | 26.7 (12/45) |
|  | CRP+ | 55.6^†^ (50/90) | 27.8 (15/54) | 36.4 (44/121) | 25.5 (13/51) |
|  | CRP− | 45.9 (34/74) | 35.1 (13/37) | 25.0 (21/84) | 25.0 (11/44) |
|  | MRI+ | 53.0^†^ (70/132) | 28.6 (20/70) | 32.8 (44/134) | 26.1 (18/69) |
|  | MRI− | 43.8 (14/32) | 38.1 (8/21) | 29.6 (21/71) | 23.1 (6/26) |
|  | HLA-B27+ | 54.9^§^ (67/122) | 33.3 (21/63) | 33.6 (44/131) | 31.8 (21/66) |
|  | HLA-B27− | 42.5 (17/40) | 23.1 (6/26) | 27.4^‡^ (20/73) | 10.3 (3/29) |
| **BASDAI50** | CRP+/MRI+ | 48.4^*^ (30/62) | 13.9 (5/36) | 40.8^§^ (20/49) | 10.5 (2/19) |
|  | CRP+/MRI− | 34.4 (11/32) | 40.0 (8/20) | 32.4 (23/71) | 16.1 (5/31) |
|  | CRP−/MRI+ | 47.1 (33/70) | 31.4 (11/35) | 24.7 (21/85) | 17.8 (8/45) |
|  | CRP+ | 47.8^§^ (43/90) | 24.1 (13/54) | 34.7^§^ (42/121) | 13.7 (7/51) |
|  | CRP− | 41.9 (31/74) | 29.7 (11/37) | 26.2 (22/84) | 18.2 (8/44) |
|  | MRI+ | 47.0^†^ (62/132) | 22.9 (16/70) | 30.6^‡^ (41/134) | 15.9 (11/69) |
|  | MRI− | 37.5 (12/32) | 38.1 (8/21) | 32.4 (23/71) | 15.4 (4/26) |
|  | HLA-B27+ | 49.2^§^ (60/122) | 30.2 (19/63) | 34.4^‡^ (45/131) | 19.7 (13/66) |
|  | HLA-B27− | 35.0 (14/40) | 15.4 (4/26) | 26.0^§^ (19/73) | 6.9 (2/29) |
| **ASAS PR** | CRP+/MRI+ | 29.0^§^ (18/62) | 8.3 (3/36) | 20.4^†^ (10/49) | 0.0 (0/19) |
|  | CRP+/MRI− | 25.0 (8/32) | 10.0 (2/20) | 18.3 (13/71) | 6.5 (2/31) |
|  | CRP−/MRI+ | 30.0^‡^ (21/70) | 11.4 (4/35) | 10.6 (9/85) | 4.4 (2/45) |
|  | CRP+ | 30.0^†^ (27/90) | 9.3 (5/54) | 19.0^†^ (23/121) | 3.9 (2/51) |
|  | CRP− | 27.0^‡^ (20/74) | 10.8 (4/37) | 10.7 (9/84) | 4.5 (2/44) |
|  | MRI+ | 28.8^†^ (38/132) | 10.0 (7/70) | 14.2^§^ (19/134) | 2.9 (2/69) |
|  | MRI− | 28.1 (9/32) | 9.5 (2/21) | 18.3 (13/71) | 7.7 (2/26) |
|  | HLA-B27+ | 33.6^†^ (41/122) | 11.1 (7/63) | 17.6^§^ (23/131) | 6.1 (4/66) |
|  | HLA-B27− | 15.0 (6/40) | 7.7 (2/26) | 12.3^§^ (9/73) | 0.0 (0/29) |
| **ASDAS-CRP ID** | CRP+/MRI+ | 32.3^*^ (20/62) | 2.8 (1/36) | 16.3^§^ (8/49) | 0.0 (0/19) |
|  | CRP+/MRI− | 18.8 (6/32) | 10.0 (2/20) | 11.3 (8/71) | 3.2 (1/31) |
|  | CRP−/MRI+ | 32.9 (23/70) | 17.1 (6/35) | 15.3 (13/85) | 11.1 (5/45) |
|  | CRP+ | 31.1^*^ (28/90) | 5.6 (3/54) | 13.2^†^ (16/121) | 2.0 (1/51) |
|  | CRP− | 28.4 (21/74) | 16.2 (6/37) | 15.5 (13/84) | 11.4 (5/44) |
|  | MRI+ | 31.8^*^ (42/132) | 10.0 (7/70) | 15.7 (21/134) | 7.2 (5/69) |
|  | MRI− | 21.9 (7/32) | 9.5 (2/21) | 11.3 (8/71) | 3.8 (1/26) |
|  | HLA-B27+ | 34.4^*^ (42/122) | 11.1 (7/63) | 15.3 (20/131) | 7.6 (5/66) |
|  | HLA-B27− | 17.5 (7/40) | 7.7 (2/26) | 12.3 (9/73) | 3.4 (1/29) |
| ^*^*P* < 0.0001, ^†^*P* < 0.001, ^§^*P* < 0.01, ^‡^*P* < 0.05 versus placebo. NRI data presented for all variables.  ASAS, Assessment of SpondyloArthritis international Society; ASDAS, Ankylosing Spondylitis Disease Activity Score; BASDAI, Bath Ankylosing Spondylitis Disease Activity Index; CRP, C-reactive protein; HLA, human leukocyte antigen; ID, inactive disease; M, number of evaluable patients; MRI, magnetic resonance imaging; NRI, non-responder imputation; PR, partial remission. | | | | | |

| **Table S3. Additional efficacy endpoints in the subgroups analysed at Week 16** | | | | |
| --- | --- | --- | --- | --- |
| **Endpoints** | **Subgroups** | | **Pooled secukinumab 150 mg** | **Placebo** |
| **BASDAI50, % responders (n/M)** | | CRP+/MRI+ | 45.0 (50/111)^*^ | 12.7 (7/55) |
|  |  | CRP+/MRI− | 33.0 (34/103) | 25.5 (13/51) |
|  |  | CRP−/MRI+ | 34.8 (54/155) | 23.8 (19/80) |
| **ASDAS-CRP ID, % responders (n/M)** | | CRP+/MRI+ | 25.2 (28/111)^*^ | 1.8 (1/55) |
|  |  | CRP+/MRI− | 13.6 (14/103) | 5.9 (3/51) |
|  |  | CRP−/MRI+ | 23.2 (36/155) | 13.8 (11/80) |
| **BASFI, LS mean change (SE) from baseline, n** | | CRP+/MRI+ | −2.60 (0.28)^*^, 107 | −0.87 (0.37), 49 |
|  |  | CRP+/MRI− | −1.57 (0.29), 100 | −1.21 (0.39), 49 |
|  |  | CRP−/MRI+ | −1.25 (0.25), 151 | −1.03 (0.32), 78 |
| ^*^*P* < 0.0001 versus placebo. NRI data presented for BASDAI50 and ASDAS-CRP ID, and MMRM data for BASFI.  ASDAS, Ankylosing Spondylitis Disease Activity Score; BASDAI, Bath Ankylosing Spondylitis Disease Activity Index; BASFI, Bath Ankylosing Spondylitis Functional Index; CRP, C-reactive protein; ID, inactive disease; LS, least squares; M, number of evaluable patients; MMRM, mixed-effects model repeated measures; MRI, magnetic resonance imaging; NRI, non-responder imputation. | | | | |
